# Correction Notice: Labeling and Isolation of Fluorouracil Tagged RNA by Cytosine Deaminase Expression

**DOI:** 10.21769/BioProtoc.3857

**Published:** 2020-12-05

**Authors:** Harihar Basnet, Joan Massague

**Affiliations:** Cancer Biology and Genetics Program, Memorial Sloan Kettering Cancer Center, 1275 York Avenue, New York, NY 10065, USA

In Procedure C, page 4 of our Bio-protocol paper, https://bio-protocol.org/e3433, we notice some mistakes. “250 µg/kg 5-FC” should be “250 mg/kg of 5-FC”. “125 µg/kg of thymine” should be “125 mg/kg of thymine”. The correct text should be:

**Day 31**

4.Inject the mice with 250 **mg**/kg 5-FC in PBS (12.5 μg/ml stock) intraperitoneally, and 125 **mg**/kg thymine in PBS (6.25 μg/ml stock) subcutaneously. 

## 
References

1. Basnet H., and Massague J. (2019). Labeling and Isolation of Fluorouracil Tagged RNA by Cytosine Deaminase Expression.
*Bio-protocol* 9 (22): e3433.

